# Ecological trade-offs between jasmonic acid-dependent direct and indirect plant defences in tritrophic interactions

**DOI:** 10.1111/j.1469-8137.2010.03491.x

**Published:** 2011-01

**Authors:** Jianing Wei, Lizhong Wang, Jiuhai Zhao, Chuanyou Li, Feng Ge, Le Kang

**Affiliations:** 1 State Key Laboratory of Integrated Management of Pest Insects and Rodents, Institute of Zoology, Chinese Academy of SciencesBeijing 100080, China; 2 State Key Laboratory of Plant Genomics and Center for Plant Gene Research, Institute of Genetics and Developmental Biology of the Chinese Academy of SciencesBeijing 100101, China

**Keywords:** ecological trade-off, genetically modified tomato plants, jasmonic acid, *Liriomyza huidobrensis*, *Opius dissitus*, plant defences, *Solanum lycopersicum*, tritrophic interactions

## Abstract

- Recent studies on plants genetically modified in jasmonic acid (JA) signalling support the hypothesis that the jasmonate family of oxylipins plays an important role in mediating direct and indirect plant defences. However, the interaction of two modes of defence in tritrophic systems is largely unknown.
- In this study, we examined the preference and performance of a herbivorous leafminer (*Liriomyza huidobrensis*) and its parasitic wasp (*Opius dissitus*) on three tomato genotypes: a wild-type (WT) plant, a JA biosynthesis (*spr2*) mutant, and a JA-overexpression *35S::prosys* plant. Their proteinase inhibitor production and volatile emission were used as direct and indirect defence factors to evaluate the responses of leafminers and parasitoids.
- Here, we show that although *spr2* mutant plants are compromised in direct defence against the larval leafminers and in attracting parasitoids, they are less attractive to adult flies compared with WT plants. Moreover, in comparison to other genotypes, the *35S::prosys* plant displays greater direct and constitutive indirect defences, but reduced success of parasitism by parasitoids.
- Taken together, these results suggest that there are distinguished ecological trade-offs between JA-dependent direct and indirect defences in genetically modified plants whose fitness should be assessed in tritrophic systems and under natural conditions.

## Introduction

The bottom-up effects of plants against herbivores indicated that plants have evolved a wide range of direct and indirect defensive strategies (Gatehouse, 2002). Plants defend themselves against insects directly through feeding (e.g. physical barriers or toxins) (Kessler & Baldwin, 2002; Peiffer *et al.*, 2009) and indirectly through attracting natural enemies of herbivores (Turlings *et al.*, 1995; Heil, 2008; Dicke *et al.*, 2009; Kang *et al.*, 2009). Both direct and indirect plant defences are constitutive and inducible, while induced strategies may be favoured over constitutive ones because of the low cost (Kessler & Baldwin, 2002). Some studies support a trade-off between direct and indirect defences because of resource limitation (Ballhorn *et al.*, 2008). For example, plant species with a strong direct defence may not invest in indirect defence through the emission of specific volatiles (Van Den Boom *et al.*, 2004). In addition, direct defence traits, for example, secondary plant compounds and physical barriers, may also have negative impacts on natural enemies of insect herbivores. However, a recent study revealed that there is no conflict between direct and indirect plant defences in a highly specialized herbivore–natural enemy system involving a brassicaceous plant species (Gols *et al.*, 2008).

To enhance plant resistance against insect pests, defence genes from plants and nonplant origins have been introduced into major crops (Schuler *et al.*, 1998; Turlings & Ton, 2006; Kos *et al.*, 2009). Although transgenic plants, such as *Bacillus thuringiensis* (Bt)-expressing crops, can effectively suppress population densities of target insect pests, concerns are often raised about the long-term and wider ecological risks associated with the release of genetically modified plants (O’Callaghan *et al.*, 2005). Rarely considered are the ecological consequences of transgenic plants on tritrophic interactions among plants, herbivorous insects and natural enemies (Groot & Dicke, 2002; Kos *et al.*, 2009). In addition, the potential effects of direct defence of genetically modified plants on the efficiency of their indirect defence, and vice versa, are not well characterized, which is probably because of the lack of genotypes that differ exclusively in the same defensive trait (Dicke *et al.*, 2004).

Tomato, *Solanum lycopersicum*, is an economically important vegetable worldwide and a commonly used model plant for biologists. It has long been used to study defence-related signalling pathways and gene expressions (Browse, 2005; Schilmiller & Howe, 2005). Accumulating evidence from tomato and other model systems supports the hypothesis that the jasmonate family of oxylipins plays an important role in mediating herbivore-triggered direct and indirect plant defences (Kessler & Baldwin, 2002; Browse, 2005). For example, impairing or silencing genes related to oxylipin signalling pathways render the mutants or transformants more susceptible to herbivores (Thaler *et al.*, 2002; Ament *et al.*, 2004; Kessler *et al.*, 2004; Li *et al.*, 2005; Chehab *et al.*, 2008; Halitschke *et al.*, 2008) and less attractive to natural enemies under both laboratory and field conditions (Thaler *et al.*, 2002; Shiojiri *et al.*,2006; Bruce *et al.*, 2008; Chehab *et al.*, 2008; Halitschke *et al.*, 2008). Similarly, genetic engineering of terpenoid metabolism or overexpression of a single gene involved in terpenoid production enables the plant to attract more natural enemies (predator, parasitic wasp or entomopathogenic nematode) (Kappers *et al.*, 2005; Schnee *et al.*, 2006; Degenhardt *et al.*, 2009). However, in most of these studies, the direct and indirect defences were investigated independently, and only a few examined their relationship in a tritrophic system, for example, a tomato, spider mite and predator mite system (Thaler *et al.*, 2002). Furthermore, the ecological consequences of genetically modified plants were rarely characterized under natural conditions (Halitschke *et al.*, 2008).

Here, we investigated the roles of the jasmonic acid (JA) pathway in regulating plant–insect interaction in a tritrophic system, including the behavioural responses of herbivore and parasitoid and the defensive signals in wild-type (WT) plants and JA mutants; in addition, the interaction of direct and indirect plant defences was analysed. We used a transgenic tomato line (*35S::prosys*) with constitutive JA signalling, and a tomato mutant with defective JA biosynthesis (*spr2*). Our study showed that, although *spr2* mutant plants are compromised in direct defence against the larval leafminers and in attracting parasitoids, they are less attractive to adult flies compared with WT plants. Moreover, in comparison to other genotypes, the *35S::prosys* plant displays greater direct and constitutive indirect defences, but reduced success of parasitism by parasitoids. These results indicate that there are distinguished ecological trade-offs between JA-dependent direct and indirect defences in tritrophic interactions under more natural conditions.

## Materials and Methods

### Plants and insects

Tomato (*Solanum lycopersicum*) line cv Castlemart was used as the WT parent for all experiments. Tomato mutant line *spr2* (Li *et al.*, 2003) was derived from cv Castlemart. Seeds for the *35S::prosystemin* transgenic plants were collected from a *35S::prosys/35S::prosys* homozygous line (Howe & Ryan, 1999) that was backcrossed five times using tomato cv Castlemart as the recurrent parent. Tomato seedlings were grown in 500 ml pots containing a mixture of peat and vermiculite (4 : 1), in environmental chambers (Conviron Co., Winnipeg, MB, Canada) under 16 h light at 28°C and 8 h dark at 18°C, with irradiance of 150 μE m^−2^ s^−1^ during photophase, and 60% relative humidity. Plants with four to six fully expanded leaves were used for experiments. In the choice experiments, the age and leaf area of each genotype were normalized by sowing in parallel under the same culture conditions.

Colonies of the pea leafminer, *Liriomyza huidobrensis*, and the parasitoid, *Opius dissitus*, were maintained as described previously (Wei & Kang, 2006). Briefly, 2-wk-old kidney bean plants (*Phaseolus vulgaris* L. cv Naibai) with two fully developed true leaves were used to culture leafminers. *O. dissitus* females emerging from pupae were mated within 24 h. All *O. dissitus* used in the behavioural assays and parasitism experiments were 2- to 4-d-old adult females without previous exposure to their host, *L. huidobrensis*, or host plants. Each female was used only once in the experiments.

### Adult leafminer preference for tomato genotypes

Feeding and oviposition preference of adult flies to intact tomato plants were monitored in a cage of 40 × 40 × 40 cm. This experiment was designed as a dual-choice test. The mutants and WT plants were of similar age and shape. For the combination of WT plant vs *spr2* mutant, two plants of each genotype were paired in the cage. Then, plants were exposed to 150–200 adult flies (male : female = 1 : 1) for 5 h under the same conditions as used for rearing the pea leafminer. For the combination of JA-overexpressing line *35S*::*prosys* vs WT plant, the number of adult leafminers or the exposure time was increased to 300 files or 7 h to obtain the comparable density of feeding and oviposition punctures on the most resistant line, the *35S*::*prosys* plant.

### Adult parasitoid preferences for tomato genotypes

#### Y-tube experiments

**Fig. 2:**
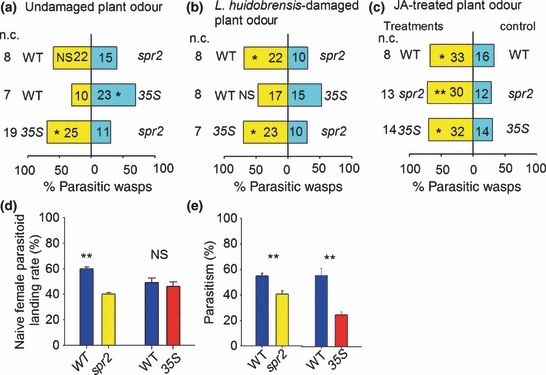
Behavioural responses and parasitism rates of larval leafminers by female *Opius dissitus* wasps. Behavioural responses of naïve female wasps to volatile blends emitted by paired undamaged tomato genotypes (a), leafminer (*Liriomyza huidobrensis*)-damaged genotypes (b) and jasmonic acid (JA)-treated genotypes (c). Bars represent the percentages of parasitoids choosing either odour source offered in a Y-tube olfactometer. Numbers in bars are the numbers of parasitoids choosing the indicated odour source. (d) Landing preference of *O. dissitus* naïve females determined as the percentage of parasitoids landing on each of the two offered tomato plant genotypes (*N*=6). (e) Larval parasitism rates (%) based on dissection of leafminer larvae 3 d after exposure to adult parasitoids (wild-type (WT) vs *spr2* plants, *N*=12; WT vs *35S::prosys* plants (*35S*), *N*=6). In each panel, *, *P* < 0.05; **, *P* < 0.01; n.c., no choice; NS, nonsignificant.

A Y-tube olfactometer was used to investigate the behavioural responses of female *O. dissitus* to the volatile blends from the various genotypes (Wei *et al.*, 2007). Each female parasitoid was placed in the olfactometer for 5 min. A ‘no choice’ outcome was recorded when the adults remained inactive during the testing period. A ‘first choice’ outcome was recorded when the adults moved > 5 cm onto either arm (visually assessed by a line marked on each arm). Each experiment involved at least 30 females making a choice. Each odour source (Fig. 2a–c) was prepared by mixing three to four volatile collections (see the Plant volatile collection section for details) and concentrating to 1000 μl in a nitrogen (purity 99.999%) atmosphere. In the dual-choice tests, each solution (10 μl) of paired genotype or treatment was applied to one piece of filter paper (1 × 2 cm), which was placed inside one of the two arms of the Y-tube olfactometer. The mean dosage of each extract was equivalent to 0.2–0.4 h entrainment of volatiles. Filter paper with plant odour was refreshed after each test.

#### Cage experiments

Landing preferences of parasitoids to the larval-damaged tomato genotypes were observed in a cage of 40 × 40 × 40 cm. This experiment was designed as a dual-choice test. Plants containing second-instar larvae were prepared as for the larval leafminer performance experiment. The mutants and WT plants used in this study were of similar age and shape, and had similar leafminer larvae numbers (Supporting Information, Table S1). Two WT tomato plants and two mutant plants, either *spr2* or *35S::prosys* containing leafminer larvae, were paired and enclosed in the cage. An individual naive female parasitoid was released at the midpoint between two groups of tomato plants and its behaviour was monitored for 10 min. The plant on which parasitoid landed and its subsequent searching attempts (e.g. walking and touching mines) were recorded as a choice. If the parasitoid did not show any oviposition attempts when landing or did not land on any plant during the test period, it was recorded as ‘no choice’. At least 30 female wasps were tested in a cage with the same set of plants. Based on the number of landing parasitoids on each tomato genotype, a landing rate (%) was calculated as the preference for this genotype. Each assay was replicated at least six times with newly prepared plants.

### Parasitism of leafminer larvae on tomato genotypes

The plant treatment and experimental design were the same as for the preference assays of parasitoid wasps. Two WT tomato plants and two mutant plants, each infested with similar numbers of leafminer larvae (Table S1), were enclosed in a cage. The number of parasitoids released into each cage was calculated to achieve a ratio of one parasitoid for every 10 larvae. Our preliminary experiments showed that no superparasitism would occur under this ratio within 24 h. Parasitism was determined by dissecting leafminer larvae 3 d after exposure to adult parasitoids when larval parasitoids had developed into the first-instar larval stage.

### Larval leafminer performance on each tomato genotype

A no-choice experiment was designed to examine larval performance on each genotype. Plant preparation and treatments were similar to experiments regarding adult leafminer preferences discussed earlier. For the JA-overexpressing line, *35S*::*prosys*, the exposure time and the number of adult leafminers were doubled to obtain similar number of larvae as other genotypes (Table S1). Subsequently, the exposed plants loaded with leafminer eggs were moved into growth chambers for development.

To determine larval survival on each genotype, plants with leafminer eggs were monitored daily until the larval mines became visible. Then the old and new mines (viable eggs) were counted and marked by a black marker to avoid recounting, usually on day 3–4 after oviposition. When larvae completed development and were ready to pupate, plants were laid down on Styrofoam trays and all larvae emerging from mines were collected. Puparia from each genotype were collected and counted daily, and were kept in glass vials (60 × 15 mm). Larval survival (%) was calculated as the ratio of total pupae number to viable eggs.

To investigate leafminer development on each genotype, plants with leafminer eggs were monitored twice daily (at *c.* 08:00 and 16:00 h) from egg hatching to pupation, and the time from pupation to adult eclosion was recorded daily. Each day, 10–15 randomly selected larvae were removed from the leaves of each plant genotype using a pin. They were preserved in 70% ethanol and the developmental stage was determined as described by Petitt (1990). Larvae numbers at each developmental stage were recorded. An estimate of larval area (length × width) was used to represent host size (Ode & Heinz, 2002). We photographed and measured larvae with a microscope (Leica DFC490; Leica, Wetzlar, Germany) and processed the images using the qwin plus software (Leica). More than 20 larvae were measured for each genotype and developmental stage. When larvae completed development and were ready to pupate, plants were laid down on Styrofoam trays and the numbers of collected larvae or pupae at each time were recorded. Puparia were collected from each genotype and placed in glass vials (60 × 15 mm), and adult emergence was monitored daily. The developmental time, defined as the required time for 50% of the population to reach a certain developmental stage, was recorded for each larval stage and tomato genotype in h, and then converted into d.

### Quantification of proteinase inhibitor (PI)-II proteins

Using a radial immunodiffusion assay described by Ryan (1967), we quantified the amounts of PI-II protein in the leaflets of each genotype. Briefly, an agar plate was prepared using 2% Noble agar (Sigma A-5431) in 0.1 M sodium veronal, 0.9% NaCl, at pH 8.5, to which 1% goat polyclonal tomato PI-II antiserum was added.

Samples in each genotype were taken from undamaged and larval leafminer-damaged tomato plants, respectively. For each undamaged tomato genotype, leaflets were removed from lower, middle and upper layers of two to three randomly selected plants and pooled into a mortar, and the leaf contents were extracted. For each leafminer-infested genotype, PI-II was measured on days 4, 5 and 7, respectively, after oviposition by adult flies as described earlier. For each replication per genotype, leaflets were removed from lower, middle and upper layers of two to three randomly selected plants, with or without leafminer damage, and pooled into a mortar, and the leaf contents were extracted. Five millilitres of leaf extract from each treatment were placed into a well (0.5 mm diameter) of agar plate. After 24 h, the diameter of the immunoprecipitate ring was measured to calculate the amount of PI-II based on the following formula: ((diameter × diameter − 625) × 0.016) (Ryan, 1967). Both local and systemic PI-II amounts were determined for leafminer-damaged plants. There were at least six replications per genotype and treatment.

### Plant volatile collection

Tomato plants for volatile collection were treated as described in *Larval leafminer performance*. For each treatment of leafminer-damaged plant, tomato plants with 4–6 expanded leaves were transferred to environmental growth chambers as described in *Plants* section. To determine the difference of JA-induced volatile emission in various tomato genotypes, tomato plant roots were placed in vials containing 50 ml of aqueous JA (Sigma-Aldrich) solution (1 mM) with 0.5% alcohol for 48 h (Hopke *et al.*, 1994), and the vials were sealed with Parafilm. Control plants were placed in vials with 50 ml of 0.5% alcohol for 48 h. JA-treated plants or controls were transferred to individual glass tubes filled with 50 ml of tap water, and then were subjected to the volatile collection system.

The headspace volatile collection system was designed as described by Wei *et al.* (2006, 2007) with minor modifications. Briefly, two plants were placed in a plastic oven bag (40 × 44 cm; Reynolds®, Richmond, Virginia, USA), into which a stream of filtered and moisturized air was pumped through two freshly activated charcoal traps. The air with emitted plant volatiles was withdrawn through a glass collector containing 100 mg Porapak Q (80–100 mesh size; Supelco, Bellefonte, PA, USA) by a membrane pump (Beijing Institute of Labor Instruments, Beijing, China) at a rate of 400 ml min^−1^ for 10 h. Five to six collections were made simultaneously, with one blank bag as control, at 24 ± 2°C and 180 μE m^−2^ s^−1^ irradiance. The absorbed volatiles from Porapak Q collectors were then extracted with 700 μl of high-performance liquid chromatography (HPLC)-grade dichloromethane (Tedia Company, Fairfield, Ohio, USA). All aeration extracts were stored at −20°C until used in chemical analyses or behavioural experiments. To quantify volatiles, plants were weighed immediately after volatile collection using an electronic balance (AE 240; Mettler, Toledo, Switzerland). The larvae numbers in tomato leaflets were recorded by carefully examining leaves under a stereo microscope (Wild, Heerbrugg, Switzerland).

### Chemical identification and quantification of collected volatiles

The chemical structures of collected volatile compounds were identified as described by Wei *et al.* (2007) with small modifications. Briefly, an Agilent gas chromatographer (GC) (6890N) coupled with a mass spectrometry (MS) system (5973 MSD; Agilent Technologies, Inc., Palo Alto, CA, USA) was equipped with either a DB-WAX column (60 m × 0.25 mm ID, 0.15 μm film thickness) or a DB5-MS column (60 m × 0.25 mm ID × 0.15 μm film thickness; Agilent Technologies) for chemical identifications. On the DB-WAX column, the oven temperature was initially kept at 40°C for 4 min and then increased by a rate of 10°C min^−1^ to 200°C, followed by a rate of 10°C min^−1^ to 230°C. The inlet was operated in the splitless injection mode, and the injector temperature was maintained at 250°C with a constant flow rate of 1.0 ml min^−1^. The GC-MS electron impact source was operated in the scan mode with the MS source temperature at 230°C and the MS Quad at 150°C. Volatile compounds were identified by comparing their retention time and spectra with synthetic standards (see Wei *et al.*, 2006; plus 2-carene, 97%; *p*-cymene, ≥ 97%; (*Z*)-3-hexenyl butyrate, ≥ 98%; Aldrich). Referenced mass spectra were from the NIST02 library (Scientific Instrument Services, Inc., Ringoes, NJ, USA).

To quantify collected volatiles, an Agilent GC (7890A) coupled with an auto-injector (7683 autoinjector module, cataloge number G2913A; Agilent Technologies, Inc.) was equipped with the DB-WAX column described earlier, and the same thermal programme was adopted. Mixed samples consisting of heptanoic acid, ethyl ester and dodecanoic acid, ethyl ester in different concentrations (1, 5, 20, 50, or 100 ng μl^−1^) were used as external standards for developing standard curves to quantify volatiles.

### Data analysis

Data were analysed using the spss software package (version 15.0; SPSS Inc., Chicago, IL, USA). In dual-choice tests, the oviposition preference of adult leafminer and parasitism and the landing rates of parasitic wasps were compared by paired *t*-test. For parametric analysis, the percentage data (survival and landing rates) were arcsin(*x*^1/2^)-transformed, while the absolute quantity data were log(*x* + 1)-transformed to correct heterogeneity of variances. Each experiment was replicated four to six times. The chi-squared test was used to determine the significance of difference between the numbers of parasitoids choosing each olfactometer arm (Wei & Kang, 2006; Wei *et al.*, 2007). Parasitoids that did not make a choice were excluded from statistical analysis. Larval survival (%) and body size were compared among three genotypes using ANOVA and Tukey’s honestly significant difference (HSD) test. Developmental time of *L. huidobrensis* was compared using a Kruskal–Wallis test followed by a Mann–Whitney *U*-test. The temporal difference of JA-regulated PI-II accumulation in three genotypes was analysed using repeated-measures ANOVA with plant genotype as an independent factor, and the PI-II amount at each observation period was compared by Kruskal–Wallis test followed by Mann–Whitney *U*-test. The amounts of volatiles released from each genotype and treatment were normalized to ng h^–1^ (10 g)^–1^ plant FW and compared by Student’s *t*-test. The amounts of leafminer-induced volatiles emitted by each genotype were normalized to ng h^–1^ per 100 larvae (10 g)^–1^ plant FW and compared by ANOVA and HSD tests (Wei *et al.*, 2006).

## Results

### Adult leafminer preferences for tomato genotypes

**Fig. 1:**
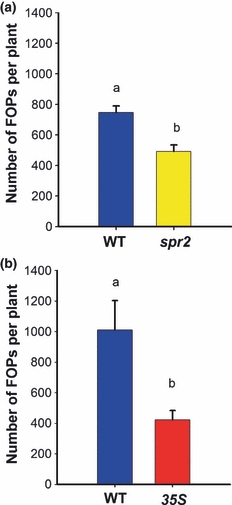
Feeding and oviposition preferences of adult leafminer *Liriomyza huidobrensis* for tomato genotypes. Mean (*N*=6) number of feeding and oviposition punctures (FOPs) of adult leafminers on paired wild-type (WT) and *spr2* plants (a), or on paired WT and *35S::prosys* (*35S*) plants (b). FOPs were compared between the plants of each pair by a paired samples *t*-test (two-tailed). Data were arcsin(*x*^1/2^)-transformed. Significant differences between two tomato genotypes are indicated by different letters on each bar (*P*<0.05).

To determine the preference of *Liriomyza huidobrensis* adult flies for different tomato genotypes, feeding and oviposition punctures (FOPs) on WT plants paired with each mutant were monitored in cages. Our results showed that the adults preferred WT plants over the JA-deficient mutant *spr2* (paired samples *t*-test, *t =*5.36, df= 5, *P*=0.003; Fig. 1a) and the JA-overexpressing genotype *35S::prosys* (paired samples *t*-test, *t =*11.21, df= 5, *P*<0.0001; Fig. 1b).

### Adult parasitoid preferences for tomato genotypes

To determine the preference of *O. dissitus* female wasps for volatiles emitted by different tomato genotypes, behavioural responses and parasitism of larval leafminers by female wasps were investigated in a dual-choice olfactometer and/or in a cage. Our results showed no difference in behavioural preference of female wasps between the undamaged WT and JA-deficient mutant plants (Fig. 2a), but the undamaged *35S::prosys* line was significantly preferred by the parasitoids over the other genotypes in the Y-tube tests (*35S::prosys* vs WT, *χ*^2^ = 5.12, *P*=0.024; *35S::prosys* vs *spr2*, *χ*^2^ = 5.44, *P*=0.02). In addition, leafminer-damaged WT and *35S::prosys* plants were significantly more attractive to parasitoids than the leafminer-damaged JA-deficient mutant (WT vs *spr2*, *χ*^2^ = 4.50, *P*=0.034; WT vs *35S::prosys*, *χ*^2^ = 0.125, *P*=0.724; *35S::prosys* vs *spr2*, *χ*^2^ = 5.12, *P*=0.024; Fig. 2b). JA-treated *spr2*, *35S::prosys*, and WT plants were all more attractive to female wasps than the controls (JA-*spr2* vs *spr2*, *χ*^2^ = 11.55, *P*=0.0098; JA-*35S::prosys* vs *35S::prosys*, *χ*^2^ = 7.04, *P*=0.0079; JA-WT vs WT, *χ*^2^ = 5.90, *P*=0.015; Fig. 2c). In cage experiments, the number of first landings for naive female parasitoids on WT plants was significantly higher than on the JA-deficient genotype (paired samples *t*-test, WT vs *spr2*, *t =*8.52, df= 5, *P*<0.001, Fig. 2d), while it was similar to that on *35S::prosys* plants (paired samples *t*-test; WT vs *35S::prosys*, *t =*0.54, df= 5, *P*=0.614; Fig. 2d).

To determine the impact of this odour-driven preference on parasitism, we measured the parasitism rates in dual-choice experiments in cages. Indeed, the parasitoid’s preference for WT plants over the JA-deficient mutant *spr2* caused a higher degree of parasitism (paired samples *t*-test, *t =*6.92, df= 11, *P*<0.0001; Fig. 2e). However, compared with *35S::prosys*, larvae on WT plants were parasitized by the parasitoids at a higher rate (*t =*5.84, df= 5, *P*=0.002; Fig. 2e).

### Direct defence: pea leafminer performance on three tomato genotypes

**Fig. 3:**
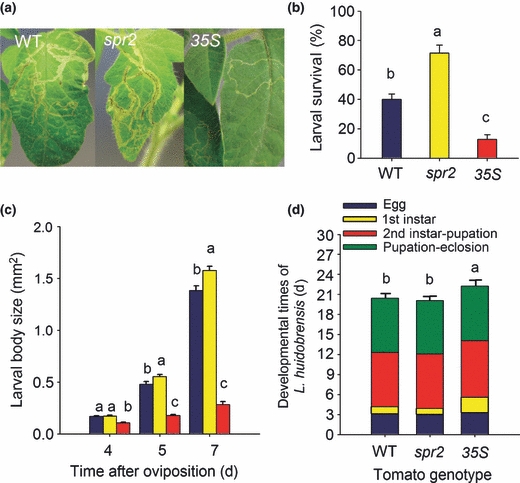
Effects of direct defence on the performance of pea leafminer, *Liriomyza huidobrensis*, in different tomato genotypes. (a) Larval mining damage on the leaflet of each genotype at day 7 (the third-instar larva stage) after female leafminer ovipositions. (b) Larval survival (%) on each genotype. The bar represents the mean values for five replicates of each genotype (*N*=5). (c) Larval body size (length × width, mm^2^) on each genotype. The bar represents five replicates of each genotype (*N*=5) (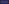, wild-type (WT); 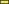, *spr2*; 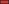, *35S::prosys* (*35S*)). Days 4, 5 and 7 after oviposition correspond to the developmental stage of first-, second-, and third-instar larvae, respectively. (d) Developmental time (d) from egg hatch to pupation was recorded for each genotype. There were at least six replications per genotype per developmental stage (*N*=6). Bars indicate means ± SEM, and significant differences among three tomato genotypes are indicated by letters on each bar.

The performance of leafminers differed significantly among three genotypes (Fig. 3a–d; larval body size on the fifth day: ANOVA, *F*_2,63_=37.60, *P* < 0.001; also see Fig. S1; developmental time from egg to adult eclosion: Kruskal–Wallis test, *χ*^2^=14.7, df= 2, *P* < 0.001; also see Fig. S2a; pupal weight: ANOVA, *F*_2,26_=122.15, *P* < 0.001; proportion of adult eclosion was similar for the three genotypes: ANOVA, *F*_2,12_=1.02, *P* = 0.335; see Fig. S2b). Larval survival (%) on WT plants was significantly lower than on the JA-defective mutant *spr2*, but it was much higher than on the JA-overexpressing genotype *35S::prosys* (ANOVA, *F*_2,12_ = 89.16, *P*<0.001; Fig. 3b). Therefore, the performance of leafminers on WT plants was poorer than on *spr2* plants but better than on *35S::prosys* plants.

### PI-II in three tomato genotypes

**Fig. 4:**
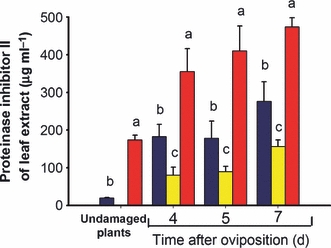
Proteinase inhibitor II (PI-II) accumulates in the leaflets of three tomato genotypes in response to leafminer damage (*N*=6). Bars indicate means ± SEM; significant differences among three tomato genotypes are indicated by letters on each bar. Days 4, 5 and 7 after oviposition correspond to the developmental stage of first-, second-, and third-instar larvae, respectively. WT, wild-type; *35S*, *35S::prosys* (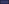, WT; 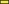, *spr2*; 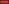, *35S*).

Accumulation of PI-II differed significantly among the three tomato genotypes (repeated measures ANOVA, *F*_genotype, 2,62_ = 127.64, *P*<0.0001; Fig. 4) and varied with time (*F*_time, 2,62_ = 58.83, *P*<0.0001; *F*_genotype × time_ = 16.92, *P*<0.001). Compared with the other two genotypes, undamaged *35S::prosys* plants constitutively expressed higher amounts of PI-II protein (Fig. 4). Leafminer infestation triggered PI-II accumulation in *spr2* plants, but the amounts were significantly lower than the accumulation in WT plants (Fig. 4). In contrast, PI-II accumulation in leafminer-treated *35S::prosys* plants was dramatically higher than in WT plants (Fig. 4). Thus, PI-II accumulation was negatively correlated with leafminer performance on the three tomato genotypes (*F*_PI-survival%, 1,12_ = 17.12, *r*^2^ = 0.633, *P* = 0.0019; *F*_PI-larval body size, 1,12_ = 29.20, *r*^2^ = 0.745, *P* < 0.0001; *F*_PI-development times, 1,12_ = 31.04, *r*^2^ = 0.756, *P* < 0.0001; Figs 3, 4).

### Indirect defence signals in three tomato genotypes

**Fig. 5:**
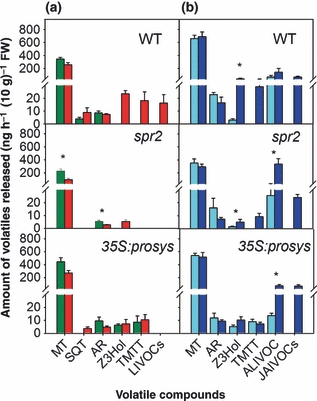
Leafminer (*Liriomyza huidobrensis*)- and jasmonic acid (JA)-induced volatile compounds released from three tomato genotypes. The volatile profiles include undamaged (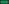) and leafminer-damaged (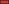) tomato plants (a), and 0.5% alcohol-treated (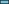) and JA-treated (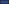) plants (b). MT, monoterpenes (α-pinene, 2-carene, α-phellandrene, limonene, β-phellandrene); SQT, sesquiterpene (β*-*caryophyllene); AR, aromatic (*p*-cymene); Z3Hol, (*Z*)-3-hexenol; TMTT, (3*E*,7*E*)-4,8,12-trimethyl-1,3,7,11-tridecatetraene; LIVOCs, other leafminer-induced volatile organic compounds ((*Z*)-3-hexenyl butyrate, (*Z*)-3-hexenyl acetate); ALIVOC, alcohol-induced volatile compounds (butanoic acid, ethyl ester); JAIVOCs, other JA-induced volatile compounds ((*E*)-β*-*ocimene, (*E*)-2-hexenal, (*Z*)-3-hexenyl acetate, propanoic acid, ethyl ester). Compound lists of tomato genotypes and treatments are presented in the Supporting Information (Tables S2, S3); comparisons among genotypes or between treatments are presented in Table S4. Bars indicate means ± SEM; *, *P <*0.05. In (a) there were five replications per genotype per treatments (*N*=5). In (b) there were four replications per genotype per treatments (*N*=4).

To investigate the role of the JA signalling pathway in regulating volatile emissions, volatile profile, an indicator of indirect defence, was examined in three tomato genotypes. Undamaged, leafminer-damaged, 0.5% alcohol-treated (JA control) and JA-treated tomato plants released five monoterpenes (MTs) and one aromatic compound (AR, *p*-cymene) (Fig. 5; for compound list see Tables S2, S3). Interestingly, the typically inducible compounds, (*Z*)-3-hexenol (Z3Hol) and (3*E*,7*E*)-4,8,12-trimethyl-1,3,7,11-tridecatetraene (TMTT), were constitutively released by the undamaged and alcohol-treated *35S::prosys* plants. However, when plants were damaged by leafminer, the JA-deficient mutant emitted significantly lower amounts of inducible volatiles [Z3Hol, TMTT and other leafminer-induced volatile compounds (LIVOCs)] in comparison to WT and *35S::prosys* plants (ANOVA, *F*_2,12_ = 14.38, *P*<0.001) and, qualitatively, WT plants released higher numbers of inducible compounds than other genotypes (Fig. 5a). Upon JA treatment, the three genotypes emitted similar amounts of inducible volatiles [Z3Hol, TMTT, and other JA-induced volatile compounds (JAIVOCs)] (ANOVA, *F*_2,9_ = 2.08, *P* = 0.11; Fig. 5b). In addition, the emission of (*Z*)-3-hexenol was substantially higher in leafminer-damaged WT plants than in the other two mutants (ANOVA, *F*_2,12_ = 6.27, *P*=0.033; Fig. 5a), with *spr2* plants and the JA-overexpressing *35S::prosys* line emitting 28.6 and 40%, respectively, of the amount emitted by WT plants. The release rate of TMTT from *35S::prosys* mutants was comparable to WT plants (*t =*1.86, df= 8, *P*= 0.465). In contrast, the amounts of (*Z*)-3-hexenol and TMTT were significantly higher in JA-treated WT plants than in other genotypes (ANOVA, *F*_2,9_ = 18.9, *P*<0.001).

## Discussion

### Ecological trade-off between direct and indirect plant defences in JA-overexpression plants

Our data showed that although the amounts of inducible compounds were significantly lower in *35S::prosys* plants than in WT plants after leafminer damage (see Fig. 5), naive female wasps did not discriminate between leafminer-damaged *35S::prosys* and WT plants, suggesting that the induced indirect defence was not affected in the JA-overexpressing line. One question is whether constitutive emission of attractants by resistant genotypes poses a conflict between direct and indirect plant defences (but see Turlings & Ton, 2006 and Gols *et al.*, 2009). Interestingly, we demonstrated in a cage experiment that larvae feeding on the JA-overexpressing line were parasitized at a lower rate than those feeding on WT plants. It is possible that plant secondary chemicals, such as toxins and anti-digestive proteins, have a negative impact on the development of herbivorous insects as well as on the fitness and/or foraging behaviour of their natural enemies (Gols *et al.*, 2009). We found that after 5 d of development, PI-II accumulation in the leaflets of *35S::prosys* plants was 2.3-fold higher than that in WT plants, and the average size of larvae on *35S::prosys* was threefold smaller than on WT plants (see Figs 3d, S1). In addition, female *O. dissitus* wasps preferred to frequently parasitize in bigger larvae than in smaller ones (Bordat *et al.*, 1995). These data imply that there is a potential ecological trade-off between direct and indirect plant defences in the JA-overexpressing tomato genotype, and the reduced parasitism on *35S::prosys* plants may be partially the result of the poor quality of host larvae (Gols *et al.*, 2009). Another study showed that JA treatment of a tomato cultivar (*Lycopersicon esculentum* var. Ace) reduced the performance of a caterpillar (*Spodoptera exigua*) and its parasitoid wasp (*Hyposoter exiguae*) (Thaler, 1999). However, this study revealed increased rates of parasitism in JA-induced plants, which were not attributed to longer parasitoid exposure or lower herbivore quality. The author postulated that increased volatile production from JA-treated plants attracted more parasitoids and resulted in a higher parasitism rate, which was inconsistent with our results using a *prosystemin* overexpression line. Based on the slow growth/high mortality hypothesis (Benrey & Denno, 1997) and the fact that the larval development was about 4 d longer (Fig. 3) on *35S::prosys* plants than on WT plants, one might predict a higher overall vulnerability to parasitism of larvae feeding on *35S::prosys* than on WT plants. Nevertheless, Benrey & Denno (1997) and Williams (1999) also indicated that slow growth does not always result in increased parasitism, especially when different host plant species with various defence traits were compared or when parasitoids are involved.

### Inducible volatiles, such as (*Z*)-3-hexenol and TMTT, play important role in host habitat location by parasitoids

Our data also demonstrated that transgenic *35S::prosys* plants have a higher degree of direct defence against the leafminers than WT plants. Since constitutive and induced expression of PI-II was enhanced in *35S::prosys* plants, it is not surprising that this genotype exhibited the highest resistance to leafminers. Interestingly, undamaged *35S::prosys* plants also constitutively released the typical defence-related volatiles, HIPVs (herbivore-induced plant volatiles), (*Z*)-3-hexenol and TMTT, all of which were undetectable in other genotypes when undamaged. Transgenic tomato overexpressing the *prosystemin* gene exhibits enhanced expression of TomLOX-C (lipoxygenase C in tomato), TomLOX-D and hydroperoxide lyase (HPL) genes (Corrado *et al.*, 2007), which could explain our finding that this JA-overexpressing mutant constitutively released (*Z*)-3-hexenol and TMTT. Behavioural assays in the Y-tube olfactometer showed that female parasitic wasps preferred the odour of undamaged *35S::prosys* plants to that of the other two genotypes (Fig. 3a), confirming that the inducible volatiles, such as (*Z*)-3-hexenol and TMTT, can effectively attract parasitic wasps to the leafminers’ habitat (Wei *et al.*, 2007). The production of saturated hexanol and hexanal was higher in *spr2* mutants than in WT and *35S::prosys* plants (Sanchez-Hernandez *et al.*, 2006), but the preference of parasitoids was not interfered with by these saturated C6 compounds.

### Ecological trade-off in herbivore attraction in JA-deficient mutants

We found that the *spr2* mutant displayed markedly reduced direct and indirect defences against larval leafminers. Susceptibility to a larval leafminer was correlated with decreased production of PI-II protein and reduced attraction and parasitism success by a parasitic wasp, which was consistent with the decreased inducible volatile emission. In herbivore-damaged plants, the reduced PI-II accumulation and volatile emission in the JA biosynthesis mutant *spr2* could be the result of the loss of function of ω-3 chloroplast fatty acid desaturase, which inhibits LA (the lipid-derived fatty acid 18:3) content in leaflets to < 10% of the amount in WT plants (Li *et al.*, 2003). Meanwhile a corresponding increase in LA proportion (18:2) resulted in augmented production of saturated hexanol and hexanal in this mutant (Li *et al.*, 2003; Sanchez-Hernandez *et al.*, 2006). Moreover, compared with WT and *35S::prosys* plants, reduced volatile emission was constitutively detected in unwounded and wounded *spr2* plants (see Table S4), which was consistent with a previous study using *spr2* plants (Sanchez-Hernandez *et al.*, 2006). The observed reduction of volatile emission in this mutant could be the result of impaired MT synthesis as a consequence of decreased expression of 1-deoxy-d-xylulose 5-phosphate synthase (*DXS2*), which is a key gene involved in volatile synthesis in chloroplast (Sanchez-Hernandez *et al.*, 2006). A green leaf volatile, (Z)-3-hexenol, was decreased in *spr2* plants to 28.6% of the amount in WT plants after leafminer damage. Interestingly, we found that this mutant is less attractive to adult flies in a cage experiment compared with WT plants (Fig. 1a), which was consistent with a study using a transgenic *Nicotiana attenuata* plant in the field (Halitschke *et al.*, 2008), suggesting that there is an ecological trade-off in terms of herbivore attraction in JA-regulated defence and that the lower attraction to foraging herbivores might be the result of the reduced emission of green leaf volatiles. However, it was also reported that *Manduca sexta* adults preferred to oviposit on *spr2* in the cage experiment (Sanchez-Hernandez *et al.*, 2006). Therefore, these plants should be examined under natural conditions to elucidate the underlying mechanisms and the corresponding ecological consequences.

In summary, based on our studies and other reports (Thaler *et al.*, 2002; Ament *et al.*, 2004; Kessler *et al.*, 2004; Halitschke *et al.*, 2008), we hypothesize that silencing of the jasmonate cascade simultaneously attenuates direct and indirect defence traits, but also makes plants less attractive to herbivores. In addition, using a transgenic plant, our study provides the first evidence that enhanced direct defence may compromise the efficiency of indirect defence. Parasitism on silenced and overexpressing genotypes was less successful than on the WT plants, irrespective of whether the genetic manipulations resulted in improved or impaired direct defence in modified plants. Therefore, there are remarkable ecological trade-offs between JA-dependent direct and indirect defences in terms of herbivore attraction and parasitoid acceptance in genetically modified plants. We do not intend to discourage efforts to enhance plant defences by using plant- and/or nonplant-originated resistance genes against agricultural insect pests. Instead, as a promising and vital component of integrated pest management, genetic engineering should consider the traits for both direct and indirect defence (Turlings & Ton, 2006; Degenhardt *et al.*, 2009; Kos *et al.*, 2009), and vice versa. In particular, the effects of direct defence on the performance of the third trophic level should not be ignored and the plant fitness should be examined under natural conditions.

## Supporting Information

**Fig. S1** Photographs of larval leafminers collected from three tomato genotypes.

**Fig. S2** *Liriomyza huidobrensis* performance on three tomato genotypes.

**Table S1** The mean number of larvae (mean ± SEM) per plant used in different experiments

**Table S2** Presence of volatile compounds released from undamaged plants (UDPs) and *L. huidobrensis* larvae-damaged plants (Lh-LDPs) of three tomato genotypes

**Table S3** Presence of volatile compounds released from 0.5% alcohol-treated plants (ALPs) and jasmonic acid-treated plants (JAPs) of three tomato genotypes

**Table S4** Statistical analysis of total volatile emissions among genotypes or between the undamaged and leafminer-infested plants, or between 0.5% alcohol-treated (jasmonic acid (JA) controls) and JA-treated tomato plants

## References

[b1] Ament K, Kant MR, Sabelis MW, Haring MA, Schuurink RC (2004). Jasmonic acid is a key regulator of spider mite-induced volatile terpenoid and methyl salicylate emission in tomato. Plant Physiology.

[b2] Ballhorn DJ, Kautz S, Lion U, Heil M (2008). Trade-offs between direct and indirect defences of lima bean (*Phaseolus lunatus*). Journal of Ecology.

[b3] Benrey B, Denno RF (1997). The slow-growth-high-mortality hypothesis: a test using the cabbage butterfly. Ecology.

[b4] Bordat D, Coly EV, Letourmy P (1995). Influence of temperature on *Opius dissitus* (Hym: Braconidae), a parasitoid of *Liriomyza trifolii* (Dipt: Agromyzidae). Entomophaga.

[b5] Browse J (2005). Jasmonate: an oxylipin signal with many roles in plants. Plant Hormones.

[b6] Bruce TJA, Matthes MC, Chamberlain K, Woodcock CM, Mohib A, Webster B, Smart LE, Birkett MA, Pickett JA, Napier JA (2008). Cis-jasmone induces *Arabidopsis* genes that affect the chemical ecology of multitrophic interactions with aphids and their parasitoids. Proceedings of the National Academy of Sciences, USA.

[b7] Chehab EW, Kaspi R, Savchenko T, Rowe H, Negre-Zakharov F, Kliebenstein D, Dehesh K (2008). Distinct roles of jasmonates and aldehydes in plant-defense responses. PLoS ONE.

[b8] Corrado G, Sasso R, Pasquariello M, Iodice L, Carretta A, Cascone P, Ariati L, Digilio M, Guerrieri E, Rao R (2007). Systemin regulates both systemic and volatile signaling in tomato plants. Journal of Chemical Ecology.

[b9] Degenhardt J, Hiltpold I, Kllner TG, Frey M, Gierl A, Gershenzon J, Hibbard BE, Ellersieck MR, Turlings TCJ (2009). Restoring a maize root signal that attracts insect-killing nematodes to control a major pest. Proceedings of the National Academy of Sciences, USA.

[b10] Dicke M, van Loon JJA, de Jong PW (2004). Ecogenomics benefits community ecology. Science.

[b11] Dicke M, van Loon JJA, Soler R (2009). Chemical complexity of volatiles from plants induced by multiple attack. Nature Chemical Biology.

[b12] Gatehouse JA (2002). Plant resistance towards insect herbivores: a dynamic interaction. New Phytologist.

[b13] Gols R, van Dam NM, Raaijmakers CE, Dicke M, Harvey JA (2009). Are population differences in plant quality reflected in the preference and performance of two endoparasitoid wasps?. Oikos.

[b14] Gols R, Witjes LMA, Loon JJAv, Posthumus MA, Dicke M, Harvey JA (2008). The effect of direct and indirect defenses in two wild brassicaceous plant species on a specialist herbivore and its gregarious endoparasitoid. Entomologia Experimentalis et Applicata.

[b15] Groot AT, Dicke M (2002). Insect-resistant transgenic plants in a multi-trophic context. Plant Journal.

[b16] Halitschke R, Stenberg JA, Kessler D, Kessler A, Baldwin IT (2008). Shared signals –‘alarm calls’ from plants increase apparency to herbivores and their enemies in nature. Ecology Letters.

[b17] Heil M (2008). Indirect defence via tritrophic interactions. New Phytologist.

[b18] Hopke J, Donath J, Blechert S, Boland W (1994). Herbivore-induced volatiles – the emission of acyclic homoterpenes from leaves of *Phaseolus lunatus* and *Zea mays* can be triggered by a *beta*-glucosidase and jasmonic acid. FEBS Letters.

[b19] Howe GA, Ryan CA (1999). Suppressors of systemin signaling identify genes in the tomato wound response pathway. Genetics.

[b20] Kang L, Chen B, Wei J-N, Liu T-X (2009). Roles of thermal adaptation and chemical ecology in *Liriomyza* distribution and control. Annual Review of Entomology.

[b21] Kappers IF, Aharoni A, van Herpen TWJM, Luckerhoff LLP, Dicke M, Bouwmeester HJ (2005). Genetic engineering of terpenoid metabolism attracts bodyguards to *Arabidopsis*. Science.

[b22] Kessler A, Baldwin IT (2002). Plant responses to insect herbivory: the emerging molecular analysis. Annual Review of Plant Biology.

[b23] Kessler A, Halitschke R, Baldwin IT (2004). Silencing the jasmonate cascade: induced plant defenses and insect populations. Science.

[b24] Kos M, van Loon JJA, Dicke M, Vet LEM (2009). Transgenic plants as vital components of integrated pest management. Trends in Biotechnology.

[b25] Li CY, Liu GH, Xu CC, Lee GI, Bauer P, Ling HQ, Ganal MW, Howe GA (2003). The tomato Suppressor of *prosystemin-mediated responses 2* gene encodes a fatty acid desaturase required for the biosynthesis of jasmonic acid and the production of a systemic wound signal for defense gene expression. Plant Cell.

[b26] Li CY, Schilmiller AL, Liu GH, Lee GI, Jayanty S, Sageman C, Vrebalov J, Giovannoni JJ, Yagi K, Kobayashi Y (2005). Role of beta-oxidation in jasmonate biosynthesis and systemic wound signaling in tomato. Plant Cell.

[b27] O’Callaghan M, Glare TR, Burgess EPJ, Malone LA (2005). Effects of plants genetically modified for insect resistance on nontarget organisms. Annual Review of Entomology.

[b28] Ode PJ, Heinz KM (2002). Host-size-dependent sex ratio theory and improving mass-reared parasitoid sex ratios. Biological Control.

[b29] Peiffer M, Tooker JF, Luthe DS, Felton GW (2009). Plants on early alert: glandular trichomes as sensors for insect herbivores. New Phytologist.

[b30] Petitt FL (1990). Distinguishing larval instars of the vegetable leafminer, *Liriomyza sativae* (Diptera, Agromyzidae). Florida Entomologist.

[b31] Ryan CA (1967). Quantitative determination of soluble cellular proteins by radial diffusion in agar gels containing antibodies. Analytical Biochemistry.

[b32] Sanchez-Hernandez C, Lopez MG, Delano-Frier JP (2006). Reduced amounts of volatile emissions in jasmonate-deficient *spr2* tomato mutants favour oviposition by insect herbivores. Plant, Cell & Environment.

[b33] Schilmiller AL, Howe GA (2005). Systemic signaling in the wound response. Current Opinion in Plant Biology.

[b34] Schnee C, Kollner TG, Held M, Turlings TCJ, Gershenzon J, Degenhardt J (2006). The products of a single maize sesquiterpene synthase form a volatile defense signal that attracts natural enemies of maize herbivores. Proceedings of the National Academy of Sciences, USA.

[b35] Schuler TH, Poppy GM, Kerry BR, Denholm I (1998). Insect-resistant transgenic plants. Trends in Biotechnology.

[b36] Shiojiri K, Kishimoto K, Ozawa R, Kugimiya S, Urashimo S, Arimura G, Horiuchi J, Nishioka T, Matsui K, Takabayashi J (2006). Changing green leaf volatile biosynthesis in plants: an approach for improving plant resistance against both herbivores and pathogens. Proceedings of the National Academy of Sciences, USA.

[b37] Thaler JS (1999). Jasmonate-inducible plant defences cause increased parasitism of herbivores. Nature.

[b38] Thaler JS, Farag MA, Pare PW, Dicke M (2002). Jasmonate-deficient plants have reduced direct and indirect defences against herbivores. Ecology Letters.

[b39] Turlings TCJ, Loughrin JH, Mccall PJ, Rose USR, Lewis WJ, Tumlinson JH (1995). How caterpillar-damaged plants protect themselves by attracting parasitic wasps. Proceedings of the National Academy of Sciences, USA.

[b40] Turlings TCJ, Ton J (2006). Exploiting scents of distress: the prospect of manipulating herbivore-induced plant odours to enhance the control of agricultural pests. Current Opinion in Plant Biology.

[b41] Van Den Boom CEM, Van Beek TA, Posthumus MA, De Groot A, Dicke M (2004). Qualitative and quantitative variation among volatile profiles induced by *Tetranychus urticae* feeding on plants from various families. Journal of Chemical Ecology.

[b42] Wei J, Wang L, Zhu J, Zhang S, Nandi OI, Kang L (2007). Plants attract parasitic wasps to defend themselves against insect pests by releasing hexenol. PLoS ONE.

[b43] Wei JN, Kang L (2006). Electrophysiological and behavioral responses of a parasitic wasp to plant volatiles induced by two leaf miner species. Chemical Senses.

[b44] Wei JN, Zhu JW, Kang L (2006). Volatiles released from bean plants in response to agromyzid flies. Planta.

[b45] Williams IS (1999). Slow-growth, high-mortality – a general hypothesis, or is it?. Ecological Entomology.

