# Supplementary material for: Ecological trade-offs between jasmonic acid-dependent direct and indirect plant defences in tritrophic interactions
Source: New Phytol. 2011 Jan;189(2):557–67. doi: 10.1111/j.1469-8137.2010.03491.x (PMC3039750; doi:10.1111/j.1469-8137.2010.03491.x)
Supplement: Supplementary file 1 [file nph0189-0557-SD1.doc]

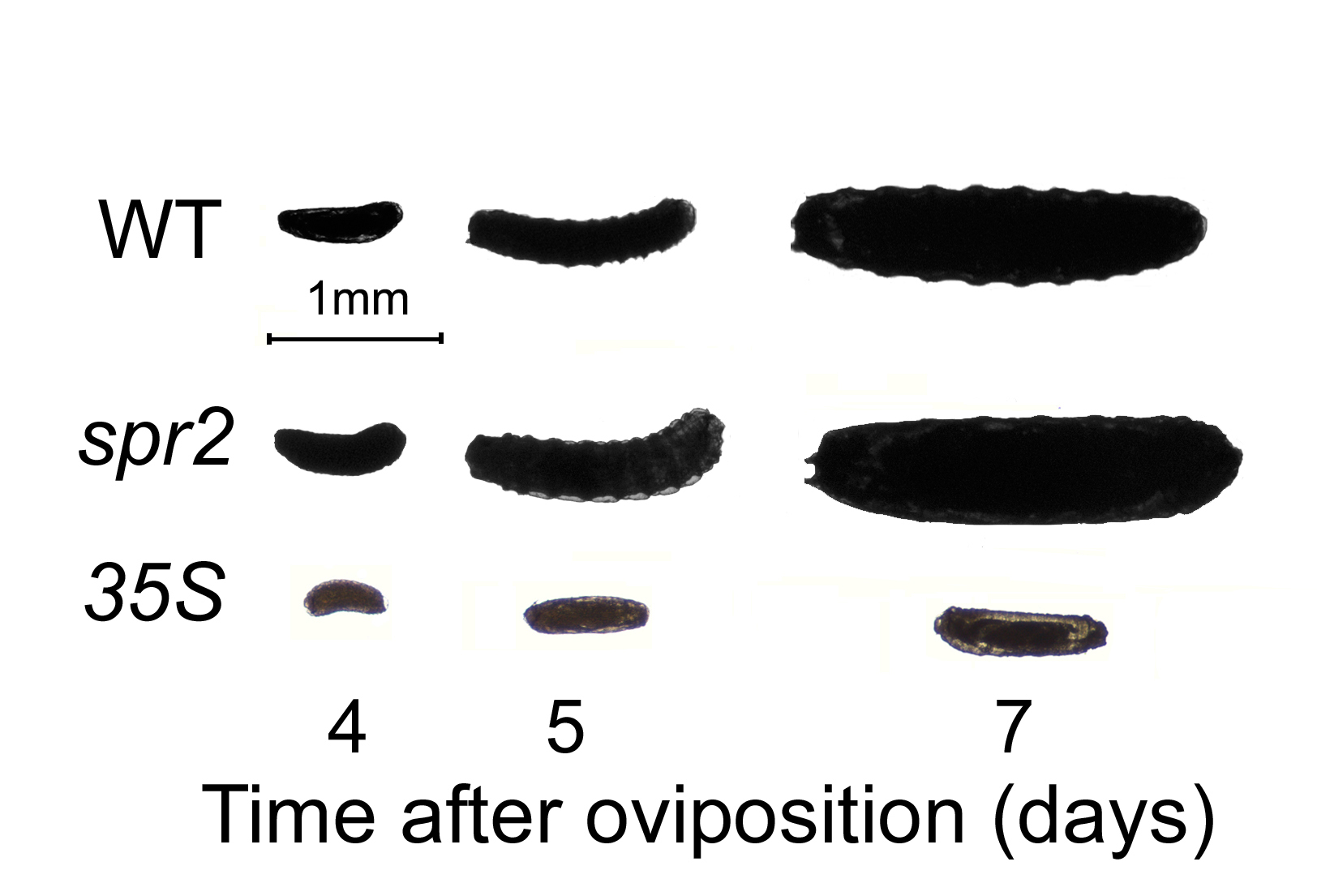


**Figure S1 Photos of larval leafminers collected from three tomato genotypes.**  The larvae collected from each genotype were measured with a microscope (LEICA DFC490) under transmitted light and were photographed by QWin plus software. Day 4, 5, and 7 after oviposition correspond to first-instar larva, second-instar larva, third-instar larva respectively. WT: wild-type; *35S* : *35S::prosys*.


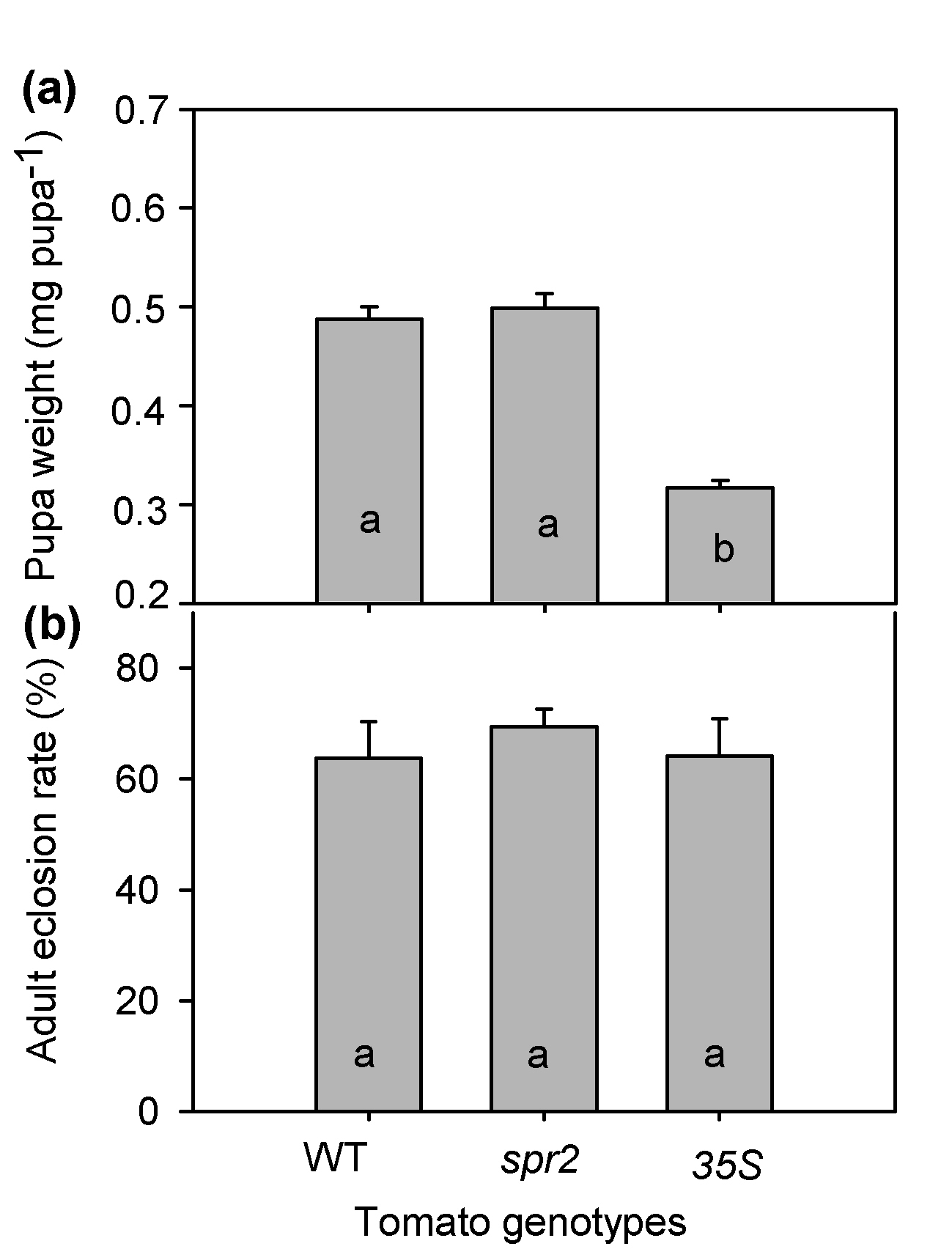


**Figure S2** ***Liriomyza huidobrensis* performance on three tomato genotypes.** The parameters include pupal weight (**a**) and adult eclosion rate (%) (**b**). In **a, b,** Significant differences among three tomato genotypes are indicated by different letters on each bar (*P* < 0.05, ANOVA follows Tukey’s honestly significant difference (HSD) for means comparison). Before parametric analysis eclosion rate was arcsine (x^1/2^) transformed, whereas pupal weight was log (x+1) transformed to correct for heterogeneity of variances. WT: wild-type; *35S* : *35S::prosys*. There were at least five replications per genotype for pupa weight or eclosion rate (*n* ≥ 5) and in each replication 20-30 pupae were measured or observed.
