# Supplementary material for: Ecological trade-offs between jasmonic acid-dependent direct and indirect plant defences in tritrophic interactions
Source: New Phytol. 2011 Jan;189(2):557–67. doi: 10.1111/j.1469-8137.2010.03491.x (PMC3039750; doi:10.1111/j.1469-8137.2010.03491.x)
Supplement: Supplementary file 2 [file nph0189-0557-SD2.doc]

**Table S1** The mean number of larvae (mean±SEM) per plant used in different experiments

| Experiments | Tomato genotypes | | | |
| --- | --- | --- | --- | --- |
|  | WT | *Spr2* | WT | *35S::prosys* |
| Landing preferences of parasitoids | 102.75±10.4*****  n = 12 | 91.1±10.8*****  n = 12 | 116.5±20.4*****  n = 12 | 83.5±17.1*****  n = 12 |
| Parasitism of leafminer larvae | 238.9±22.4***** n = 24 | 199.7±21.3***** n = 24 | 149.8±20.7*****  n = 12 | 120.2±21.2*****  n = 12 |
| Larval leafminer performance | 153.4±15.5^†^  n = 22 | 172.5± 31.1^†^  n = 16 |  | 157.3±12.9^†^  n = 16 |

^*^ The mean number of the second-instar larvae per plant in two-choice tests. n denotes replicates.

^†^ The mean number of the first-instar larvae per plant in no-choice tests.

| **Table S2 Presence of volatile compounds released from undamaged plants (UDP) and *L. huidobrensis* larvae-damaged plants (Lh-LDP) of three tomato genotypes**. | | | | | | | | |
| --- | --- | --- | --- | --- | --- | --- | --- | --- |
| **Chemical compound^*^** | LRI ^†^ | Tomato genotypes, status, and presence and absence of chemicals | | | | | | Group^§^ |
|  |  | WT | | *spr2* | | *35S::prosys* | |  |
|  |  | UDP | Lh-LDP | UDP | Lh-LDP | UDP | Lh-LDP |  |
| *α*-Pinene | 970 | √^‡^ | √ | √ | √ | √ | √ | MT |
| 2-Carene | 1085 | √ | √ | √ | √ | √ | √ | MT |
| Limonene | 1129 | √ | √ | √ | √ | √ | √ | MT |
| *α*-Phellandrene | 1141 | √ | √ | √ | √ | √ | √ | MT |
| *β*-Phellandrene | 1148 | √ | √ | √ | √ | √ | √ | MT |
| *p*-Cymene | 1242.3 | √ | √ | √ | √ | √ | √ | AR |
| (*Z*)-3-Hexenyl acetate | 1242.6 |  | √ |  |  |  |  | LIVOCs |
| (*Z*)-3-Hexenol | 1338 |  | √ |  | √ | √ | √ | Z3Hol^¶^ |
| (*Z*)-3-Hexenyl butyrate | 1402 |  | √ |  |  |  |  | LIVOCs |
| *β-*Caryophyllene | 1555 | √ | √ |  |  |  | √ | SQT |
| TMTT ^¶^ | 1771 |  | √ |  |  | √ | √ | TMTT |

***** Volatiles present at 0.1% or higher proportions in the headspace samples are listed in the table.

^†^ LRI: Linear retention index. The retention times obtained from an alkane-mixture (AccuStandard, New Haven, USA), which were analyzed under the same GC-MS condition as were used for the headspace samples, were used to calculate LRI of each compound (van den Dool and Kratz, 1963).

^‡^ “√” signifies that the volatile compound was present in the corresponding headspace sample.

^§^ Group: MT, monoterpenes; SQT, sesquiterpene; AR, aromatic; LIVOCs, other leafminer-induced volatile organic compounds.;

^¶^ Z3Hol: (*Z*)-3-Hexenol; TMTT: (3*E*,7*E*)-4,8,12-trimethyl-1,3,7,11-tridecatetraene.

Reference

**van den Dool H, Kratz PD. 1963.** A generalization of retention index system including linear temperature programmed gas-liquid partition chromatography. *Journal of Chromatography* **11**: 463-471.

| **Table S3 Presence of volatile compounds released from 0.5% alcohol-treated plants (ALP) and jasmonic acid-treated plants (JAP) of three tomato genotypes**. | | | | | | | | |
| --- | --- | --- | --- | --- | --- | --- | --- | --- |
| **Chemical compound^*^** | LRI^†^ | Tomato genotypes, status, and presence and absence of chemicals | | | | | | |
|  |  | WT | | *spr2* | | *35S::prosys* | | Group^§^ |
|  |  | ALP | JAP | ALP | JAP | ALP | JAP |  |
| Propanoic acid, ethyl ester | 917 |  | √ |  | √ |  | √ | JAIVOCs |
| *α*-Pinene | 970 | √ | √ | √ | √ | √ | √ | MT |
| Butanoic acid, ethyl ester | 989 | √ | √ | √ | √ | √ | √ | ALIVOC |
| 2-Carene | 1085 | √ | √ | √ | √ | √ | √ | MT |
| Limonene | 1129 | √ | √ | √ | √ | √ | √ | MT |
| *α*-Phellandrene | 1141 | √ | √ | √ | √ | √ | √ | MT |
| *β*-Phellandrene | 1148 | √ | √ | √ | √ | √ | √ | MT |
| (*E*)-2-Hexenal | 1159 |  | √ |  |  | √ | √ | JAIVOCs |
| (*E*)-*β*-Ocimene | 1199 |  | √ |  | √ |  | √ | JAIVOCs |
| *p*-Cymene | 1242.3 | √ | √ | √ | √ | √ | √ | AR |
| (*Z*)-3-Hexenyl acetate | 1242.6 |  | √ |  | √ |  | √ | JAIVOCs |
| (*Z*)-3-Hexenol | 1338 | √ | √ | √ | √ | √ | √ | Z3Hol |
| TMTT ^¶^ | 1771 |  | √ |  | √ | √ | √ | TMTT |

***** Volatiles present at 0.1% or higher proportions in the headspace samples are listed in the table.

^†^ LRI: Linear retention index.

^‡^ “√” signifies that the volatile compound was present in the corresponding headspace sample.

^§^ Group: MT, monoterpenes; SQT, sesquiterpene; AR, aromatic; LIVOCs, other leafminer-induced volatile organic compounds.;

^¶^ Z3Hol: (*Z*)-3-Hexenol; TMTT: (3*E*,7*E*)-4,8,12-trimethyl-1,3,7,11-tridecatetraene.

**Table S4. Statistical analysis of total volatile emissions among genotypes or between the undamaged and leafminer-infested plants, or between 0.5% alcohol-treated (JA control) and JA-treated tomato plants**

| Genotypes and treatments ^*^ | *df* | *F or t-*value | P-value |
| --- | --- | --- | --- |
| Among undamaged plants | 2, 12 | *F =* 7.042 | 0.0095** ^†^ |
| Among insect-damaged plants | 2, 12 | *F =* 23.93 | 0.000064*** |
| Among 0.5% alcohol-treated plants | 2, 10 | *F*= 26.37 | 0.00067*** |
| Among JA-treated plants | 2, 10 | *F*= 47.21 | 0.000037*** |
| Undamaged versus leafminer-damaged plants |  |  |  |
| WT | 8 | *t* = 2.25 | 0.098 |
| *35S::prosys* | 8 | *t* = 0.11 | 0.443 |
| *spr2* | 8 | *t* = 3.31 | 0.021* |
| 0.5% alcohol-treated versus JA-treated plants |  |  |  |
| WT | 6 | *t* = 3.402 | 0.027* |
| *35S::prosys* | 6 | *t* = 3.038 | 0.038* |
| *spr2* | 6 | *t* = 13.93 | 0.00015*** |

^*^ The total amounts of volatile emissions from tomato genotypes were compared among genotypes or treatments by ANOVA followed by Tukey’s honestly significant difference (HSD) for means comparison among means and between treatments by independent Student’s *t*-test (two tailed). Before parametric analysis, data were log (x+1) transformed to correct for heterogeneity of variances.

^†^ Asterisk : * *P <* 0*.*05; ** *P <* 0*.*01; *** *P <* 0*.*001.
